# Current environmental status of the oyster farms on Lake Kamo in Japan; viral control of the harmful bloom of *Heterocapsa circularisquama*

**DOI:** 10.7717/peerj.14813

**Published:** 2023-05-11

**Authors:** Natsuko Nakayama, Saho Kitatsuji, Masami Hamaguchi

**Affiliations:** 1 Fisheries Technology Institute, Japan Fisheries Research and Education Agency, Hatsukaichi, Hiroshima, Japan; 2 Faculty of Marine Science and Technology, Research Center for Marine Bioresources, Fukui Prefectural University, Obama, Fukui, Japan

**Keywords:** Lake Kamo, Enclosed, Low inflow, Viral control, Heterocapsa circularisquama bloom, HcRNAV

## Abstract

Lake Kamo is an enclosed, low-inflow estuary connected to the open sea that is famous for oyster farming in Japan. In the fall of 2009, this lake experienced its first bloom of the dinoflagellate *Heterocapsa circularisquama*, which selectively kills bivalve mollusks. This species has been detected exclusively in southwestern Japan. The completely unexpected outbreak of *H. circularisquama* in the northern region is believed to have been caused by the contamination of purchased seedlings with this species. The water quality and nutrient data collected by our group from July through October over the past 10 years revealed that the environment of Lake Kamo has not changed significantly. However, in the open water around Sado Island, where Lake Kamo is located, the water temperature has increased by 1.80 °C in the last 100 years, which is equivalent to 2–3-fold the world average. This has resulted in a rise in the sea level, which is expected to further deteriorate the water exchange between Lake Kamo and the open sea and low dissolved oxygen in the bottom layer of the Lake and the associated dissolution of nutrients from the bottom sediment. Therefore, seawater exchange has become insufficient and the lake has become nutrient rich, making it prone to the establishment of microorganisms, such as *H. circularisquama*, once they have been introduced. We developed a method to mitigate the damage caused by the bloom by spraying sediments containing the *H. circularisquama* RNA virus (HcRNAV), which infects *H. circularisquama*. After ∼10 years of performing various verification tests, including field trials, this method was used at the Lake in 2019. During the 2019 *H. circularisquama* growth season, a small amount of sediment containing HcRNAV was sprayed on the lake three times, which resulted in a decrease in *H. circularisquama* and an increase in HcRNAV, indicating that this method is effective in diminishing the bloom.

## Introduction

Global warming has changed the areas where harmful algae appear, sometimes causing large-scale damage. The marine dinoflagellate *Heterocapsa circularisquama* Horiguchi, which is one of the most harmful microalgae, forms harmful algal blooms (HABs) that specifically kill bivalves (Horiguchi, 1995; Matsuyama, 1999; Matsuyama, 2003). Although the blooms caused by *H. circularisquama* were observed in Tolo Harbor, Hong Kong, in 1986 and 1987 (Iwataki, Wong & Fukuyo, 2002), in Japan, the species were discovered in 1987 in Uranouchi Bay, Kochi prefecture. Since then, the species have frequently formed blooms, mainly in western Japan. However, the distribution area of *H. circularisquama* is expanding, and the occurrence of *H. circularisquama* has been recently observed in areas of northern Japan.

Lake Kamo is a saltwater lake where oysters have been cultivated since the early 1900s. Oysters from the lake are one of Sado’s best-known products, which are famous for ecotourism in Japan. However, the lake experienced its first bloom of the dinoflagellate *H. circularisquama* in the fall of 2009. Operations of oyster culturing in the lake suffered serious economic losses, that were estimated at over US $2 million (Kondo et al., 2012). Since then, *H. circularisquama* blooms have been detected on a yearly basis. In the past, negative effects of HABs have been reported in Japan, mainly on the southwestern coast; however, in recent years, the area of HAB outbreak area has gradually expanded toward the northeast (Kondo et al., 2012). Global warming has increased water temperatures, thereby allowing species that have been introduced to the area to grow in high-latitude waters (Kondo et al., 2012). Lake Kamo is an enclosed lake with poor water circulation, and once species, such as dinoflagellates, attach to cultured oysters and pearl oyster seedlings (Rosa et al., 2013), they easily become established. Hence, the development of measures to prevent expansion of HABs and their effective mitigation has been highly desirable in Japan.

Studies have been conducted to reduce the effects of HABs using physical (Kim, 2006; Shirota, 1989), chemical (Li et al., 2014; Sun et al., 2004a; Sun et al., 2004b; Yu et al., 2017), and biological (Marcoval et al., 2013; Tang et al., 2015; Yang et al., 2015) methods over the past several decades (Imai, Inaba & Yamamoto, 2021). However, field trials allowing the practical mitigation of blooms have been very limited because of high cost, potential negative effects on the ecosystem, and difficulties in implementation under field conditions.

The *H. circularisquama* RNA virus (HcRNAV) can infect *H. circularisquama* (Tomaru et al., 2004; Nakayama et al., 2013a) and is considered the major natural agent controlling the population dynamics of *H. circularisquama* in the environment (Tomaru et al., 2007). HcRNAV is a single-stranded RNA virus with a length of approximately 4.4 kb and contains two open reading frames, with the upstream ORF1 encoding a polyprotein involved in replication and the downstream ORF2 encoding the major capsid protein, of approximately 38 kDa (Tomaru et al., 2004). Ongoing routine monitoring has demonstrated that HcRNAV propagates during the declining phase of the blooms of *H. circularisquama* at sea, followed by virus accumulation in the sediment (Tomaru et al., 2007; Nakayama & Hamaguchi, 2016).

We have been developing a biological control method using HcRNAV, which is present in marine sediments. We hypothesized that the use of sediments with algicidal activity could provide an effective and ecofriendly method to protect bivalves, such as cultured oysters, by reducing *H. circularisquama* blooms in the natural environment. In this context, viruses exhibit host specificity and have high replication rates; therefore, co-occurring organisms suffer limited effects because a single algal species can be targeted, and viruses are effective even when used at low levels (Nakayama et al., 2020). However, spreading the virus itself in the environment was not allowed because of the negative image of the “virus”. Therefore, we decided to use sediment with accumulation of HcRNAV. This method involves dispersing marine sediments containing HcRNAV collected at the site of the bloom into the early stages of the *H. circularisquama* bloom (Nakayama et al., 2013b; Nakayama et al., 2020). This method is associated with a high degree of safety coupled with a low impact on the ecosystem and has low cost (Nakayama et al., 2013b; Nakayama et al., 2020).

Here, we report environmental data pertaining to water temperature, nutrients, and other parameters for the last 10 years in Lake Kamo. Moreover we will discuss how *H. circularisquama* was accidentally introduced and how it proliferated in the lake each summer and how conditions, such as poor closed water exchange, suits the bloom. In addition, we report on whether the “viral control” strategy developed to mitigate the damage caused by the *H. circularisquama* bloom using autochthonous HcRNAV-containing sediments suppressed the bloom.

## Materials & Methods

### Monitoring the environmental conditions at Lake Kamo over 10 years

**Figure 1:**
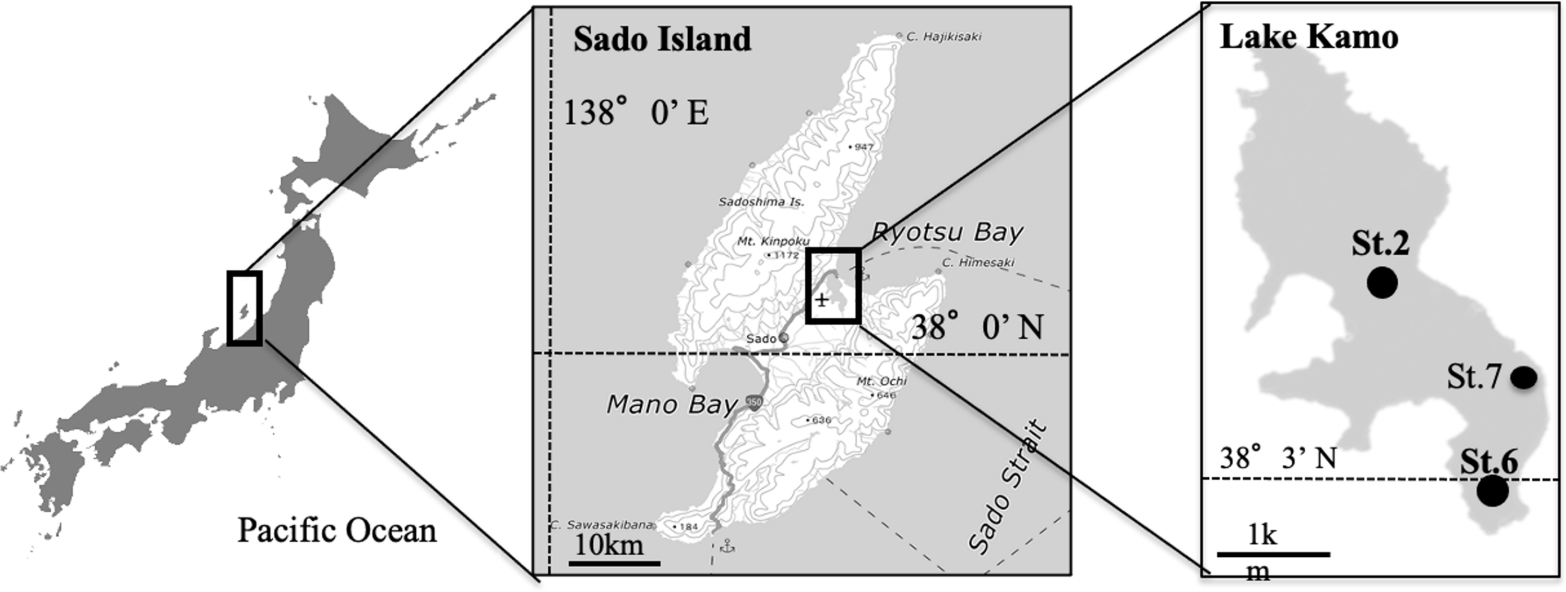
The location of the study site in Lake Kamo. The field investigation and the field trial were conducted at Station (St.) 2, 6, 7.

Monitoring and sampling were performed once every 2 weeks from July to October at three sites (St.2, St.6, and St.7) in Lake Kamo (38°03′32″N latitude to 138°26′39″E longitude) (Fig. 1). Lake Kamo is a saltwater lake on Sado Island, Niigata Prefecture, and has a shore length of 16.95 km, an area of 4.95 km^2^, and a maximum depth of 9.7 m. The Lake was originally a freshwater lake with four rivers flowing into it. However, ∼120 years ago, a channel was excavated to connect it to the open sea; thus, it became a saltwater lake. Nevertheless, the channel is small, with a width of 28 m, a length of 200 m, and an average depth of 1.7 m, leading to poor seawater exchange.

Lake water was collected from depths of 0 m (the surface) and 1 m above the bottom of the lake (B-1 m) using a bucket and a Kitahara water-sampling bottle (Rigosha & Co., Ltd., Saitama, Japan). Water temperature and dissolved oxygen were measured using a Portable Dissolved Oxygen Meter (HI 9146N; Hanna Instruments JAPAN, Chiba, Japan). Salinity was measured with a Portable Electrical Conductivity Meter DKKCM-31P (TOA DKK, Tokyo, Japan) and the calibration was performed manually before each survey in accordance with the manufacturer’s instructions. The number of *H. circularisquama* cells in one mL of unfixed water sample was counted under 100-fold magnification using a light microscope (Eclipse E600; Nikon Co., Tokyo, Japan) immediately after sampling. A glass slide with a boundary line was used for the count (MATSUNAMI, Osaka, Japan). The remaining samples were kept at −20 °C in the laboratory until HcRNAV enumeration. The amount of HcRNAV was quantified by the MPN assay up to 2015 (see ‘MPN Assay’) and by mRT-qPCR after 2016, as reported by Nakayama & Hamaguchi (2016) (see ‘RNA Extraction and cDNA Preparation’ and ‘Quantitation of HcRNAV by mRT-qPCR’).

Sediment samples were collected from the surface layer (the upper ∼30 mm) using an Ekman–Birge grab and maintained at −20 °C until the experiment was performed. The subsequent series of steps, such as RNA extraction and cDNA synthesis, were processed within 6 months after the samples were frozen.

For analysis, 10 mL of the sample was filtered with a Membrane Filter Unit Dismic® Series, with a pore size of 0.22 µm (Advantec Toyo Kaisha, Ltd., Tokyo, Japan) to determine the nutrient levels in the water, and kept at −20 °C until the measurements were performed. The concentrations of dissolved inorganic nitrogen (DIN; nitrate, nitrite, and ammonium), dissolved inorganic phosphorus (DIP), and dissolved silicon (DSi) in the water samples were measured using an AutoAnalyzer (QuAAtro 2-HR; BL TEC K.K., Osaka, Japan).

### Sediment dispersal application in Lake Kamo

#### Collection of sediment for dispersal

The sediment used in this study was collected from the thin surface layer (upper ∼30 mm) using an Ekman–Birge grab at St. 7 in Lake Kamo (Fig. 1) in November of 2018. Approximately 10 kg of sediment was collected. The station is located at a site where *H. circularisquama* blooms occur frequently and HcRNAV accumulation is expected. The sediment was stored at −20 °C until the spraying was carried out, which killed harmful and toxic algal cysts.

#### Sediment dispersal at Lake Kamo in 2019

Three field demonstration tests were conducted on natural *H. circularisquama* populations to demonstrate the effectiveness of the spraying of sediment containing HcRNAV in 2011, 2016, 2018. In 2019, a pilot spreading of sediment containing HcRNAV was conducted at Lake Kamo, to check each of the work procedures in the field. Sediment was collected and frozen the previous year. First, approximately 5 kg (wet weight) of the sediment was naturally thawed at room temperature the day before the test. On the day of application, approximately 5 L of lake water containing *H. circularisquama* was collected in a bucket, and the thawed bottom sediment was added and stirred well. After incubation for ∼3 h, we traveled to a fixed point using a research boat and sprayed three locations in the order of St. 6, 2, and 7. For spraying, a 10-L bucket was used, and seawater containing sediment was poured into the bucket while moving slowly in the research boat. Sediment spraying was conducted on July 30, August 16, and September 25, 2019. Regarding the evaluation of sediment dispersal, usual monitoring was performed, *i.e.,* by comparing the density change of *H. circularisquama* and HcRNAV with past data. *H. circularisquama* was quantified by direct counting under a light microscope (see ‘Monitoring the Environmental Conditions at Lake Kamo Over 10 Years’) immediately after sampling, and HcRNAV was assessed using mRT-qPCR (Nakayama & Hamaguchi, 2016) (see ‘Quantification of HcRNAV’). Water temperature, salinity, dissolved oxygen, and nutrient levels were analyzed (see ‘Monitoring the Environmental Conditions at Lake Kamo Over 10 Years’).

### Quantification of HcRNAV

HcRNAV was quantified using the MPN method from 2010 to 2017 and *mRT-qPCR* after 2018.

#### RNA extraction and cDNA preparation

For RNA extraction from water samples, aqueous samples (10 mL) were centrifuged at 100,000 × *g* for 1.5 h, to collect viral particles. The pellet was resuspended in 250 µL of PBS buffer and RNA extraction was performed using the RNeasy Plant Mini Kit (Qiagen, Valencia, CA, USA), according to the manufacturer’s instructions.

For RNA extraction from sediments samples, 2 g of wet weight from each sediment sample was used for RNA extraction using the RNA PowerSoil Total RNA Isolation Kit (Qiagen, CA, USA), in accordance with the manufacturer’s instructions.

The purified RNA was reverse transcribed using the ReverTra Ace^®^ qPCR RT Master Mix with gDNA remover (Toyobo Co. Ltd., Life Science Department, Osaka, Japan), as per the manufacturer’s instructions. The concentration of the extracted RNA to be used for cDNA synthesis was confirmed using a Bio Spec-nano spectrophotometer (Simazu, Kyoto, Japan), as the kits require an RNA range between 0.5 pg and 0.5 µg for efficient cDNA synthesis.

#### Quantitation of HcRNAV by mRT-qPCR

The density of HcRNAV was quantified, as described by Nakayama & Hamaguchi (2016). Specifically, HcRNAV amplification was performed using multiplex real-time reverse transcription PCR (mRT-qPCR) in a final volume of 10 µL, containing 0.3 µL of the two primer sets (300 nM final concentration), 0.2 µL of the two dual-labeled probes (200 nM final concentration), 1 µL of the cDNA template, 2.4 µL of sterilized ultrapure water, and 5 µL of SSoFast probes Spermix (Bio-Rad, Hercules, CA, USA). Amplification reactions in this study were performed using the CFX touch Real-Time PCR System (Bio-Rad, Hercules, CA, USA). The thermal cycling conditions consisted of 2 min at 95 °C, followed by 45 cycles at 95 °C for 5 s and 66 °C for 10 s.

#### MPN assay

Seawater samples were mixed well and serially diluted from 10^0^–10^−8^ in SWM3 medium. Subsequently, aliquots were added to eight wells of a 96-well microtiter plate containing exponentially growing specific host algae, as described below. The plates were incubated for 7 d and checked daily for cell lysis (Nagasaki & Yamaguchi, 1997). The dilution of the culture wells that exhibited cell lysis was scored and the most probable number of infectious viral units was calculated using the BASIC program (Nishihara, Kurano & Shinoda, 1986). In this study, three *H. circularisquama* strains were used as hosts: HU9433-P (isolated from Uranouchi Bay, Kochi prefecture, Japan), HCLG-1 (from Gokashi Bay, Mie prefecture, Japan), and 05HC06 (from Ago Bay, Mie prefecture, Japan). The algal cells were incubated in a modified SWM3 medium enriched with 2 nM Na_2_SeO_3_ (Chen, Edelstein & McLachlan, 1969; Itoh & Imai, 1987; Imai et al., 1996) at 20 °C under a 12-h light–dark cycle. Light (130–150 mmol of photons m^−2^ s^−1^) was provided by cool white fluorescent illumination.

### Statistical analysis

The relationship between water temperature, dissolved oxygen, salinity, DIN, DIP, and DSi concentrations and the occurrence of *H. circularisquama* in Lake Kamo was examined using data obtained from 2009 to 2021. ANOVA and GLM analyses were performed for the aforementioned measurements in years when *H. circularisquama* occurred, as well as in other years, in addition to simple correlation analysis. The analyses were performed using the SPSS computer software (SPSS version 23, IBM, New York, USA).

## Results

### Environmental conditions and *H. circularisquama* features in Lake Kamo over the past 10 years

#### Population dynamics of *H. circularisquama* and HcRNAV from 2010 to 2020

**Figure 2:**
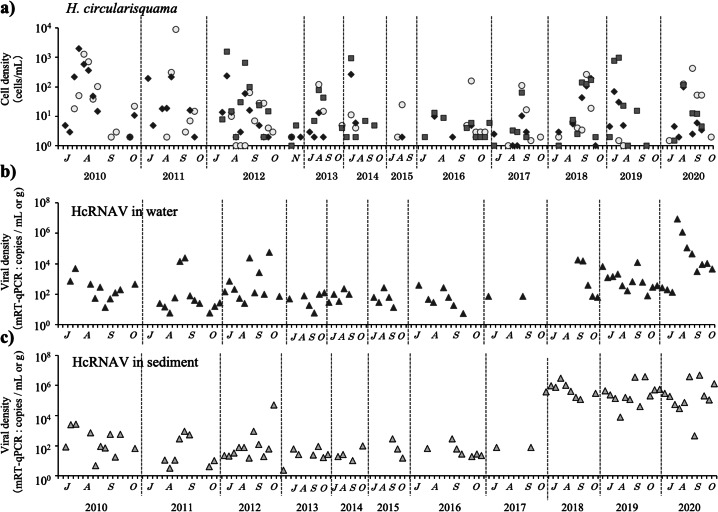
The temporal changes recorded from July to October in 2010–2020 for the cell density of *H. circularisquama* cell density (cells mL^−1^) (A) are indicated by closed circles (0 m), closed squares (6 m), and closed diamond shapes (7.3 m), and HcRANV maximum density in water (MPN method: infectious units mL^−1^ from 2010 to 2017, mRT-qPCR: copies mL^−1^ since 2018) (B), HcRNAV in sediment (MPN method: infectious units g^−1^ from 2010 to 2017, mRT-qPCR: copies g^−1^ since 2018) (C) is indicated by closed triangles.

*H. circularisquama* was first observed in Lake Kamo in October 2009, and has been occurring annually in the lake since *H. circularisquama* emerges around June or July, proliferates in the lower layers (below 6 m), reaches one or two growth peaks, and is usually undetectable by early November; moreover, it has not been detected from December to May. Two patterns of *H. circularisquama* proliferation were observed (Fig. 2A): a common pattern with peaks in July or August, and a late-autumn pattern with peaks in September or October. The *H. circularisquama* outbreaks recorded in Lake Kamo over the past 10 years were of the common type until 2015 and of the late-fall type since 2016, with the exception of 2019. Based on Figs. 2B and 2C, HcRNAVs are expected to proliferate synchronously with the emergence of *H. circularisquama* each year, repeatedly infecting and accumulating on the surface sediment. The increase or decrease in HcRNAV observed during this period reflects the presence of the host *H. circularisquama*. No significant correlation was observed during this period.

#### Water temperature, salinity, and dissolved oxygen from 2010 to 2020

### Water temperature

**Figure 3:**
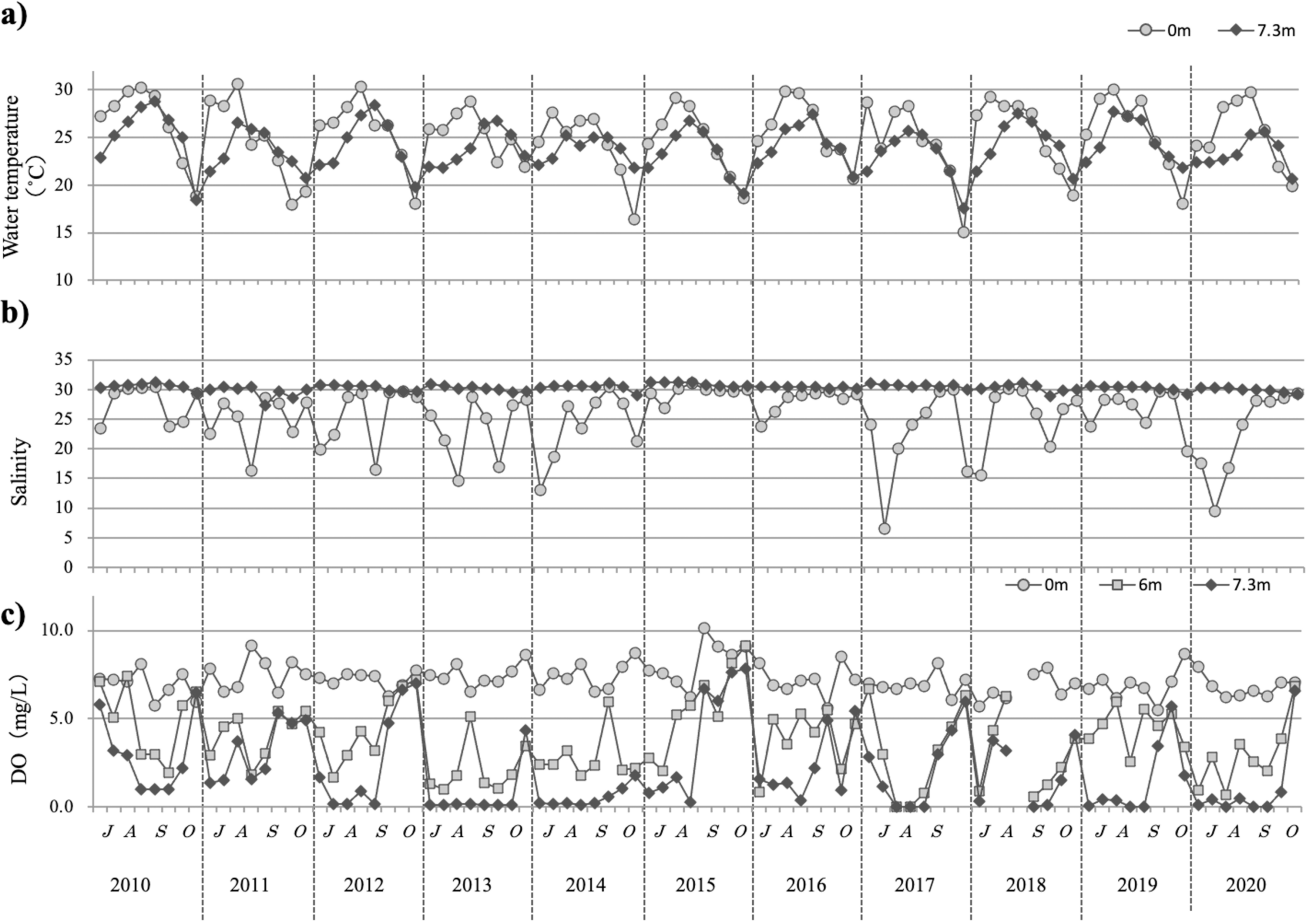
The temporal changes recorded from July to October in 2010–2020 for water temperature (A), salinity (B), and dissolved oxygen (C) are indicated by closed circles (0 m) and closed diamond shapes (7.3 m), respectively.

For about 10 years, only slight changes were observed in the water temperature from July to October (Fig. 3A). During this period, water temperature remained between 20 °C and 30 °C each year, with the highest temperature recorded at ∼30 °C and the lowest temperature dropping to ∼15 °C in some years, but staying generally at ∼20 °C. The highest water temperatures were detected generally at the surface in August, whereas deep-water temperature peaks tended to be observed ∼1 week after the surface peaks.

### Salinity

Throughout the study decade, surface salinity remained stable at ∼30 below a depth of 3 m, although it was sometimes lower in July or August because of rainfall (Fig. 3B).

### Dissolved oxygen

Almost every year for 10 years, a low oxygen blow at 4 mg/L was observed at depths >6 m from July to September, with a tendency to increase starting in mid-October. Regarding the lower levels, there were often periods of dissolved oxygen levels close to 0 mg/L. In 2015, DO was >5 mg/L in the 6 m-deep layer from early August and in the bottom layer from mid-August. In 2015, the DO was >5 mg/L in the 6 m-deep layer from early August and in the bottom layer from mid-August (Fig. 3C).

No significant differences were found for each environmental factor during this period.

#### DIN, DIP, and DSi from 2013 to 2020

**Figure 4:**
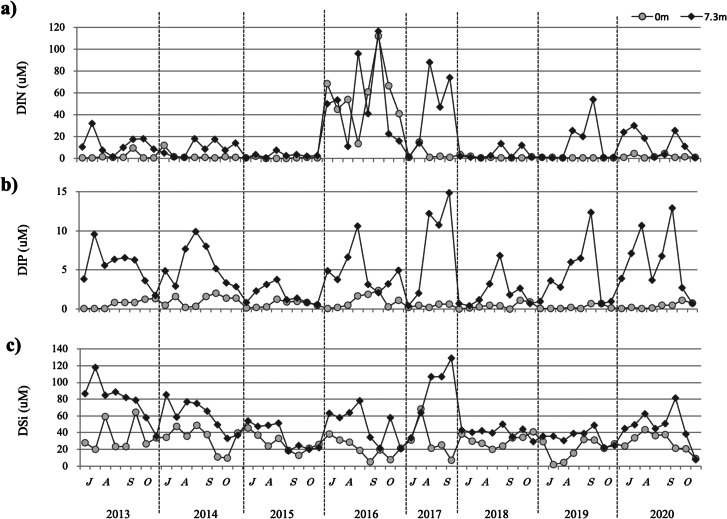
The temporal changes recorded from July to October in 2013–2020 for dissolved inorganic nitrogen DIN (A), dissolved inorganic phosphorus DIP (B), and dissolved silicate DSi (C) are indicated by closed circles (0 m) and closed diamond shapes (7.3 m), respectively.

From 2013 to 2020, the DIN remained at relatively low concentrations of 0.5–12 µM at the surface, with the exception of 2016 (Fig. 4A). In the lower layers, DIN varied yearly, although it was generally high, with the exception of 2015. In 2016, DIN was high in both the surface and bottom layers. Conversely, in 2015, the DIN was low in both the surface and bottom layers.

DIP ranged from 0.04 to 2.4 at the surface, with several periods of higher concentrations observed between July and October (Fig. 4B). DIP concentrations in the lower layers were variable, but generally high. In 2015, DIP was lower than that recorded in other years: in July and August, it was higher in the bottom layer and in September and October it was ∼1 µM in the surface layer.

DSi was generally high, but the surface layer may have been heavily affected by nutrient supply from rainfall and nutrient input from the river. It was inferred that the bottom layer is affected by nutrient dissolution because of lower DO, as observed in 2013 and 2014. In 2015, recorded DSi was relatively low compared with other years, especially in September and October. *H. circularisquama* density was low in 2015, a year in which low nutrient concentrations were recorded (Fig. 4C).

No significant differences were detected for each nutrient during this period.

### Sediment dispersal application in Lake Kamo in 2019

#### Trial of spreading sediment containing HcRNAV; population dynamics of *H. circularisquama* and HcRNAV in 2019

**Figure 5:**
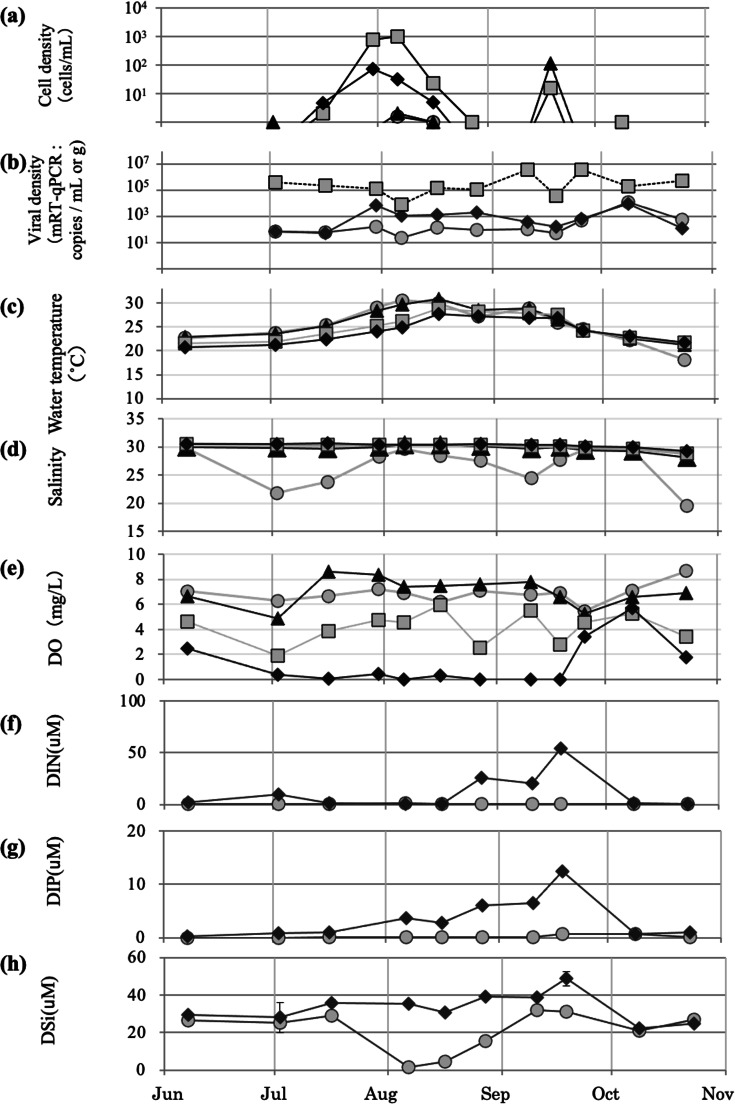
Temporary changes recorded during implementation of the 2019 viral control method. Temporal changes recorded in 2019 regarding the cell density of *H. circularisquama* (A), HcRANV density (B), water temperature (C), salinity (D), dissolved oxygen (E), dissolved inorganic nitrogen DIN (F), dissolved inorganic phosphorus DIP (G), and dissolved silicate DSi (H). Parameters recorded at 0, 3, 6, and B-1 m (approximately 7.3 m) in the lake water and sediment are indicated by closed circles (0 m), closed triangles (3 m), closed squares (6 m), and closed diamond shapes (7.3 m), respectively.

In 2019, the first detection of *H. circularisquama* was performed on June 7, with a cell density of ∼1 cell/mL; this was earlier than that recorded in previous years (Fig. 5A). The sediment was sprayed on July 30, August 16, and September 25 at St. 6, 2, and 7 in that order. An increase in *H. circularisquama* cell density was observed throughout Lake Kamo from the beginning of July to August 6, with the highest cell densities of 977 cells/mL detected at St. 2 and 919 cells/mL detected at St. 3 on August 6. The sediment was applied on July 30 and August 6, and a decrease in *H. circularisquama* and an increase in HcRNAV were observed, with *H. circularisquama* ending definitely by early September. *H. circularisquama* began to increase again from September 10 to 18. On September 10, *H. circularisquama* began to increase again; thus, the third sediment application was performed. Since then, a decrease in *H. circularisquama* and increases in HcRNAV in the seawater and sediment were observed (Figs. 5A and 5B).

#### Water temperature, salinity, and dissolved oxygen in 2019

In 2019, water temperatures rose to 20 °C–30 °C from June to early August, with the maximum water temperatures exceeding 30 °C. The temperature slowly decreased after mid-August; moreover, even after October, it only dropped to ∼22 °C, with the exception of the water surface (Fig. 5C). Salinity was affected by rainfall in the surface layer, but was stable at ∼30 at depths <3 m (Fig. 5D). Dissolved oxygen remained <2 mg/L in the bottom layer for a relatively long period, from early June to early September (Fig. 5E).

#### DIN, DIP, and DSi in 2019

DIN was low until mid-August, then increased from late August to mid-September, and decreased again in October (Fig. 5F). DIP was high from its peak in early August through September 18, but decreased again in October (Fig. 5G). DSi was low at the surface on August 6, and gradually recovered by early September (Fig. 5H).

## Discussion

*H. circularisquama* was probably introduced into Lake Kamo by accident, most likely attached to oyster or clam seedlings (Rosa et al., 2013); however, after the first bloom in 2009, it occurred every summer, and identifying the factors that cause these blooms has been challenging.

Because of rising water temperatures caused by global warming, HABs that occurred frequently in southwestern Japan have been occurring in the northeastern part of Japan for the last 10 years, and the area of HABs occurrence is steadily expanding. As discussed below, the 10-year data show no significant difference; however, the Japan Meteorological Agency reports that on the western side of the Japanese mainland, where Sado Island is located, the average water temperature has increased by 1.80 °C over 100 years. This value is approximately twice the average for the entire world (0.56 °C increase in 100 years).

Lake Kamo is in-flowed by four rivers and is connected to the open sea by a channel (Fig. 1). However, Lake Kamo is a closed saltwater lake with poor seawater exchange because it has a narrow and shallow mouth and great depth, with its maximum depth being greater than the depth of the lake mouth, with a difference of ∼6 m. In addition, water levels are increasing on the western side of the Japanese mainland as water temperatures rise. These recent conditions and the bowl-shaped geometry of Lake Kamo are considered to further prevent water from leaving the open sea, thereby interfering with water exchange in Lake Kamo.

Moreover, it is believed that most of the harmful algae that bloom in the summer cannot do so over winter because the area around 38°N, where Lake Kamo is located, is very cold during this season. *H. circularisquama* reportedly cannot grow at temperatures <10 °C and may overwinter by forming temporary cysts (Matsuyama, 2003). Conversely, seasonal water temperatures over the past 100 years were +2.45 °C from January to March, +1.87 °C from April to June, +0.99 °C from July to September, and +2.03 °C from October to December, indicating that water temperatures are particularly elevated during the winter from October to March. This suggests that *H. circularisquama* in Lake Kamo overwintered as temporary cysts or vegetative cells, and then proliferated in summer, thus becoming established in Lake Kamo. Hence, Lake Kamo became a stable system with warmer temperature and less current throughout the year, which allowed plankton to thrive.

In response to the new HABs that have emerged because of this situation, we are developing and implementing a mitigation strategy to promote the termination of the *H. circularisquama* bloom using viral control, which is a naturally occurring phenomenon.

### Relationship between *H. circularisquama* and environmental factors over the last decade

Over the approximately 10-year period from 2012 to 2020, no significant changes in water temperature were observed, although seasonal variations were detected each year. In addition, these data were compared to 10 years of data for July, August, September, and October, and no significant changes were observed over the decade. Compared with the water temperature data obtained during the environmental survey of Lake Kamo for 4 years since 2000, the water temperature recorded in August was ∼30 °C, which was not much different from the current temperature (Fig. 2A). Moreover, salinity did not vary much over the study decade, with a temporary decrease in salinity at the surface caused by rainfall; nevertheless, salinity remained stable at ∼30 at depths >3 m. As data from the past 10 years have indicated, the water temperature and salinity of Lake Kamo are stable, rendering it an environment in which microorganisms can easily proliferate. The value of DO in Lake Kamo is low, <4 mg/L, at depths >6 m, which could be a negative environment for phytoplankton growth. However, *H. circularisquama* can survive in low oxygen conditions (unpublished); in fact, the bottom layer was highly dense at the beginning of the proliferation period, in July (Fig. 2A). This may explain the dominance of *H. circularisquama* dominance observed in Lake Kamo, as it was an unfavorable environment for the growth of diatoms and other competing organisms. In addition, *H. circularisquama* can survive under poor oxygen conditions.

Nutrients were sufficient for phytoplankton growth, although there were seasonal variations. Among phytoplanktons, *H. circularisquama* has a high capacity to adapt to low nutrient concentrations (Yamaguchi, Itakura & Uchida, 2001) and can grow even under low nutrient conditions. In this study, there was no correlation between nutrient concentration and the growth of *H. circularisquama*.

During the 10-year study period, 2015 was an exceptional year. In this year, the density of *H. circularisquama* was low, nutrient concentrations were low, and the DO in the layer >6 m increased starting in August. The decrease in DSi detected starting in late August may have suppressed the growth of *H. circularisquama* because of the proliferation of competing species, such as diatoms, which have become more suited to the environment due to increase in DO. In 2016, salinity remained stable, indicating that the decrease in DSi observed in September was most likely caused by diatom growth, whereas the increase in DSi detected in October indicates a decrease in diatoms. Therefore, the sharp decrease in DO recorded at the beginning of October suggests that diatoms could no longer survive and that *H. circularisquama*, which can grow under low oxygen conditions, became dominant. In October 2016, the highest cell density of *H. circularisquama* at the observation site was ca. 160 cells/mL, whereas widespread blooms were occurring throughout Lake Kamo at depths >3 m, with the maximum cell density being approximately 4000 cells/mL (data not shown).

Closed saltwater lakes, such as Lake Kamo, where seawater exchange is slow and water temperature and salinity are stable, are considered suitable for the growth of microorganisms such as *H. circularisquama*. Furthermore, when water conditions, including low oxygen, are unfavorable for competing species, such as diatoms, it is likely that *H. circularisquama* would become dominant, *i.e.,* the relationship between the poor oxygenation derived from the closed conditions of the Lake and competing species has a significant impact on the dominance of *H. circularisquama*, which was artificially introduced into the Lake through the transfer of oyster seedlings, and on the constancy of the blooms of this species.

### Viral control of *H. circularisquama* in Lake Kamo

Although challenges remain, we have proposed a biological control method using algal viruses contained in autochthonous sediments. The basic method of sediment spreading used here was as follows. Sediment with HcRNAV accumulation was collected and frozen. This approach can maintain HcRNAV without losing infectivity while killing harmful and toxic algal cysts in the sediment. When a *H. circularisquama* bloom occurred, the seawater was collected in a small container, such as a bucket, and the sediment with HcRNAV was thawed and added to the seawater, followed by incubation for over 3 h. Subsequently, the HcRNAVs in the sediment began to infect *H. circularisquama*, *i.e.,* this seawater with the sediment included a mixture of HcRNAV-infected *H. circularisquama* and HcRNAV-containing sediments, which can be spread to increase the virus. As a result, HcRNAVs in the seawater serially infected *H. circularisquama*, eventually leading to the decline of the *H. circularisquama* bloom. This method was effective and safe and had low cost in that it used autochthonous sediment containing enormous amounts of HcRNAV and artificially accelerated natural events. In general, several issues need to be addressed before biological methods can be applied practically. Previous studies have revealed the following issues: the HcRNAV-containing sediment is sufficiently infectious, the frozen bottom sediment remains infectious for at least 3 years (Hata et al., 2012), the amount of sediment to be sprayed should be very small (ca. 0.1 g L^−1^), the timing of spraying should be more effective when *H. circularisquama* density is low, and the spreading of autochthonous sediment is harmless to other aquatic organisms (*e.g.*, oysters, clams, and other bivalve mollusks) (Nakayama et al., 2013b; Nakayama et al., 2020). Based on these results, the local government and fishermen approved the practical application of this method, which was implemented at Lake Kamo in 2019.

In the first trial, the sediment with HcRNAV was applied three times between July and October 2019. The best method is to apply the sediment to the initial outbreak area during the early stages of *H. circularisquama* development, to suppress its proliferation. In the case of Lake Kamo, St. 6, located at the inner end of the lake, is the initial outbreak point of *H. circularisquama*; St. 2 is the deepest point and easily anoxic; and St. 7 is an area where plankton gathers easily because of the wind; thus, spraying at these three points is efficient. In addition, it is important for the fishermen in the field to be able to handle it easily. In other words, efficiency must be improved. The two-step inoculation method used in this mitigation study (spraying with increasing amounts of HcRNAV) can be effective because it minimizes the amount of sediment with HcRNAV that needs to be collected and spread (Nakayama et al., 2020).

The application of 2019 was performed when the density of *H. circularisquama* was increasing, with the density of *H. circularisquama* decreasing after two sprayings. The alga began to proliferate again in the fall, but *H. circularisquama* was terminated after the third application (Figs. 5A and 5B). In addition to sediment application, HcRNAVs originally present and HcRNAVs supplied to the lake water and sediment surface after sediment application may have contributed to the decline of *H. circularisquama*.

The quantification of HcRNAVs in the environment remains a challenge. The method for quantifying HcRNAV has improved dramatically with the development of quantitative PCR (Nakayama & Hamaguchi, 2016). This is apparent in the quantitative values of HcRNAV in lake water and the sediment since 2018. However, real-time HcRNAV fluctuations are difficult to explain, because viruses are characterized by a latency period, *i.e.,* the time taken by a viral particle to reproduce inside an infected cell, during which changes in viral concentration will not be detectable.

Regarding water temperature, it is unlikely that low temperature promoted the termination of the blooms in Lake Kamo, because *H. circularisquama* blooms have been occurring during the low water temperature season in the fall in many parts of Japan (Fig. 2A), and blooms have also been occurring in Lake Kamo since 2015. Dissolved oxygen has a tendency to promote the proliferation of *H. circularisquama* at lower concentrations, according to past monitoring results. In this study, *H. circularisquama* proliferation was also observed at a lower DO at depths >6 m. Because our preliminary experiments showed that *H. circularisquama* strains can grow under low oxygen concentrations (unpublished), it is likely that, when the lower layers of Lake Kamo become anoxic, competing species cannot survive, and *H. circularisquama* can proliferate and become dominant.

Regarding nutrients, *H. circularisquama* can grow at much lower DIN and DIP based on past emergence trends in western Japan. Yamaguchi, Itakura & Uchida (2001) calculated the Q value (minimum cell quota) and Ks value (half-saturation constant of growth rate) for DIN and DIP using *H. circularisquama* cultures and found that it had the lowest values among dinoflagellates. This indicates that *H. circularisquama* has a greater ability to adapt to environments with lower nutrient concentrations compared with other phytoplankton species. These observations suggest that the contribution of HcRNAV to *H. circularisquama* decline is relatively large.

For this viral control method to be sustainable in the field, the process needs to be simple and efficient for fishermen. The spraying performed in 2020 was improved by the fishermen. As a preliminary preparation, sediment containing HcRNAV was mixed with seawater containing *H. circularisquama* bloom before spraying, to allow for the propagation of HcRNAV, The use of a polyethylene tank allowed the omission of the steps of transferring between containers and mixing with a stick to prepare for spraying. Furthermore, preparation in polyethylene tanks facilitated the transport and spread of seawater with sediment containing HcRNAV.

Even in field tests, there were concerns about the environmental impact of sediment dispersal during the practical application of this method. During the dispersal performed in 2019, approximately 1.5 kg per station was spread., At St.2, St.6, and St.7 the dispersal was performed simultaneously. For comparison, the clay dosage used at aquaculture sites in Japan was 110–400 t km^−2^ (Shirota, 1989), whereas 384 t km^−2^ of clay was used to control *Cochlodinium polykrikoides* blooms in Korea (Anderson et al., 2001). Compared with these values, the amount of sediment spread using our method was extremely small. Furthermore, as expected, the spreading of the sediment did not affect nutrient concentrations. This indicates that the two-step inoculation method of the sediment and its spraying during the initial outbreak and in areas where plankton gathered was effective in mitigation. Therefore, the effect of the dispersed sediment was considered negligible.

This viral control method was finally put into practical use in 2019. Together with previous results, we showed that it is possible to control *H. circularisquama* more easily and successfully by spreading frozen sediment with accumulated HcRNAV produced in Lake Kamo several times from the early stage of *H. circularisquama* development. Although it is difficult to rigorously evaluate the effectiveness of field methods because it is not possible to set up controls, the effectiveness and efficiency of the method could be improved by considering the relationship between HcRNAV in nature and environmental factors, such as DO and competitors, and the characteristics of HcRNAV when spraying sediments.

## Conclusions

Long-term increases in water temperature and water level in ambient waters connected to Lake Kamo have modified the lake, which is enclosed and has a poor seawater circulation rate. Hence, the lake has become stagnant, its DO has decreased, and nutrients have become concentrated. Although there were no significant correlations between the occurrence of *H. circularisquama* and environmental factors, the results of this study suggest that low oxygen conditions are suitable for the growth of *H. circularisquama* and that *H. circularisquama* is more likely to dominate because of a decrease in competing species, which may have contributed to the establishment of *H. circularisquama*. Under these circumstances, we have established and implemented a method to reduce the damage caused by the *H. circularisquama* bloom by spraying sediments containing HcRNAV, which infects and kills this dinoflagellate. During the 2019 *H. circularisquama* growth season, a small amount of sediment containing HcRNAV was dispersed in Lake Kamo, which resulted in a decrease in *H. circularisquama* density. The synchronous increase in HcRNAV indicated that this method was effective in diminishing the blooms in the field.

##  Supplemental Information

10.7717/peerj.14813/supp-1 Supplemental Information 1 Dataset of the temporal changes recorded from July to October in 2010–2020 for the density of *H. circularisquama and* HcRNAV The density of *H. circularisquama* and HcRNAV from July to October in 2010–2020.

10.7717/peerj.14813/supp-2 Supplemental Information 2 Raw dataset of the water temperature, salinity, and dissolved oxygen from July to October in 2010–2020 The temporal changes recorded from July to October in 2010–2020 for water temperature, salinity, and dissolved oxygen

10.7717/peerj.14813/supp-3 Supplemental Information 3 Raw dataset of DIN, SIP and DSi from July to October in 2013–2020 The temporal changes recorded from July to October in 2013–2020 for DIN, SIP and DSi

## Additional Information and Declarations

### Data Availability

The following information was supplied regarding data availability: The raw measurements are available in the Supplementary Files.
